# Aqueous humor perturbations in chronic smokers: a proteomic study

**DOI:** 10.1038/s41598-024-62039-6

**Published:** 2024-05-17

**Authors:** Radgonde Amer, Adi Koriat

**Affiliations:** 1 Department of Ophthalmology, Hadassah Medical Center, Jerusalem, Israel; 2 Faculty of Medicine, Hebrew University of Jerusalem, Jerusalem, Israel

**Keywords:** Health occupations, Medical research

## Abstract

The detrimental effects of smoking are multisystemic and its effects on the eye health are significant. Smoking is a strong risk factor for age-related nuclear cataract, age-related macular degeneration, glaucoma, delayed corneal epithelial healing and increased risk of cystoid macular edema in patients with intermediate uveitis among others. We aimed to characterize the aqueous humor (AH) proteome in chronic smokers to gain insight into its perturbations and to identify potential biomarkers for smoking-associated ocular pathologies. Compared to the control group, chronic smokers displayed 67 (37 upregulated, 30 downregulated) differentially expressed proteins (DEPs). Analysis of DEPs from the biological point of view revealed that they were proteins involved in complement activation, lymphocyte mediated immunity, innate immune response, cellular oxidant detoxification, bicarbonate transport and platelet degranulation. From the molecular function point of view, DEPs were involved in oxygen binding, oxygen carrier activity, hemoglobin binding, peptidase/endopeptidase/cysteine-type endopeptidase inhibitory activity. Several of the upregulated proteins were acute phase reactant proteins such as clusterin, alpha-2-HS-glycoprotein, fibrinogen, alpha-1-antitrypsin, C4b-binding protein and serum amyloid A-2. Further research should confirm if these proteins might serve as biomarkers or therapeutic target for smoking-associated ocular diseases.

## Introduction

The tobacco epidemic is a major public health danger, resulting in the death of around 8 million individuals per year globally^1^. All tobacco forms pose harm and there is no safe exposure level. Worldwide, cigarette smoking is the most common form. Cigarette smoking is one of the 10 greatest contributors to global death and disease^2^. The detrimental effects of smoking are not only local but also systemic. Smoking is linked to an increased risk of pulmonary and extrapulmonary diseases such as chronic obstructive pulmonary disease (COPD), lung cancer, diabetes mellitus, cardiovascular disease and bladder cancer^3–7^. Observational studies have associated smoking with end-products of hematopoiesis^8–14^ and hematologic neoplasms^15–17^.

Tobacco smoke contains > 4000 compounds, most of which result in carcinogenic and mutagenic activity^18^. The classes of components present in the cigarette smoke (nitrosamines, alkenes, aromatic and heterocyclic carbons and amines) are sources of reactive oxygen species (ROS)^19,20^. Besides the exogenous source of ROS, endogenous production of ROS is also enhanced in smokers^21–23^. The main effect of higher levels of ROS on cell biology is increased oxidative DNA damage^23–28^ with subsequent increase in DNA repair activity^26^. This DNA damage is an important factor in cancer development^26,29,30^.

Oxidative stress plays an important role in cataract development. The ocular lens is susceptible to oxidative insult through photocatalytic generation of oxygen radicals. The decrease in the lens antioxidant capacity causes conformational changes in lens crystallin proteins that gradually aggregate and form insoluble turbid proteins and eventually lens opacity^31^.

The effect of tobacco smoking on the eye health is significant. Smoking is a strong risk factor for age-related nuclear cataract^32^. Current smoking and a greater number of pack-years smoked increases the risk of the progression of age-related macular degeneration (AMD)^33^. Current smoking is also significantly associated with the risk of developing glaucoma and this association is even stronger among heavy smokers^34^. Current smokers are more likely to experience progression of thyroid eye disease or poorer outcome of treatment^35^. Smoking is also associated with hyperopia, delayed corneal epithelial healing and progression of Fuchs’ endothelial corneal dystrophy. Smoking during pregnancy increases the risk of convergent or divergent strabismus or poor stereo acuity^36^. Smoking was also reported to increase the likelihood that any given patient will develop uveitis^37^. Active uveitis once developed was more likely to be both more difficult to control and associated with increased risk of complications, such as cystoid macular edema^38,39^.

Focusing on the molecular basis of diseases is important in order to identify biomarkers for diagnosis and as therapeutic targets. Mass spectrometry (MS)-based proteomics has provided a means for global proteome characterization of the human fluids, including analysis of ocular fluids in different eye conditions such as cataract^40^, idiopathic epiretinal membranes^41^, rhegmatogenous retinal detachment with proliferative vitreoretinopathy^42^, neovascular AMD^43,44^, and diabetic retinopathy (DR)^45^.

Here, we aimed to analyze the aqueous humor (AH) proteome of individuals who were current smokers and to compare it to that of healthy non-smokers. Our results provide additional evidence on the perturbations of the human AH proteome of chronic smokers and an extended view on the major dysregulated pathways.

## Methods

### Subjects

All study subjects were recruited among patients who underwent elective cataract surgery. Signed informed consent was obtained from each subject prior to participation. The study was approved by the local ethics committee of Hadassah Medical Organization (0489-13-HMO) and adhered to the tenets of the Declaration of Helsinki.

Twelve patients were enrolled to this single-centered study and were included in data analysis. The study included seven healthy never-smokers and five chronic smokers.

All subjects met the inclusion criteria of no ocular disease other than cataract and no recent intraocular operation and no ocular medications other than lubricants. Exclusion criteria included systemic illnesses (such as diabetes mellitus, rheumatoid arthritis, renal failure and ischemic heart disease) and the use of systemic antimetabolites, immunosuppressants or corticosteroids.

### Aqueous humor sample collection

AH was collected from patients undergoing elective cataract surgery. Collection of AH was performed by the same operator. Right after disinfection and draping and placement of eye speculum, a clear corneal incision was made with a 1.3 mm MVR blade. The AH was then collected under the surgical microscope using a 1 mL tuberculin syringe connected to a cannula. AH was slowly aspirated until the anterior chamber began to shallow. The AH samples were rapidly cooled in ice and stored at − 80 °C before screening.

### Sample preparation

40 µL of the AH were diluted with 80 µL of 8M Urea in 25 mM Ammonium bicarbonate, pH 8.2, then reduced with 5 µL of 200 mM of dithiothreitol (DTT) to a final concentration of 8 mM (56 °C for 30 min). It was thereafter modified with 20 µL of 200 mM of 2-Iodoacetamide (IAA) to a final concentration of 27.6 mM (1 h, dark, RT). Trypsin (Promega) was added to the denatured AH at a protein to enzyme ratio of 1:20 (w/w). Trypsin digestion was carried out at 37 °C overnight. The digest was supplemented with the same amount of trypsin the following day and incubated for an additional 4 h. The tryptic peptides were desalted using C18 ZipTips (Millipore). First, the activation of the ZipTip was performed by using 100% acetonitrile (ACN) (repeated 5 times). Then it was equilibrated with 1% formic acid (FA) (repeated 5 times). Thereafter, the sample was loaded on the ZipTip. The column was again washed with 1% (v/v) FA (repeated 5 times). After that, it was eluted with ACN:DDW FA (1%) (60:40). It was then dried and resuspended in 0.1% (v/v) FA.

### Mass spectrometry analysis

The peptides were resuspended in 0.1% formic acid and 1 µg per sample was injected to LC–MS/MS analysis**.** The peptides were resolved by reverse-phase chromatography on 0.075 × 180-mm fused silica capillaries (J&W) and packed with reprosil reversed phase material (Dr. Maisch GmbH, Germany). The peptides were eluted with a linear gradient of 5–28% for 180 min, 28–95% for 15 min and for 25 min at 95% acetonitrile with 0.1% formic acid in water, at a flow rate of 0.15 μL/min. Mass spectrometry was performed by Q Exactive HF mass spectrometer (Thermo Electron, MA, USA) in a positive mode with RF level of 55. Survey scans were: range m/z 300–1800, AGC target 3e6, MAX injection time 20ms, and resolution 120,000. MS/MS analysis was performed on top 30 ions (charge states + 2 to + 7, peptide preferred option used, dynamic exclusion 20 s). Collision energy was set to 27 and MS2 scans were performed with AGC set to 1e5, max injection time 60 ms and resolution 15,000.

Data was analyzed using proteome discoverer 2.3 (SEQUEST) (Thermo Fisher Scientific Inc., USA) against human protein database from UniProt. FDR cutoff was 0.01.

### Proteome data filtering

Contaminant proteins (identified only by site and/or reverse sequence database) were excluded.

In order to acquire informative results, the proteins that were included had a peptide value greater than 2. To eliminate background bias, we only included proteins that had abundance ratio between the P-value of smokers and controls greater than 2 or smaller than 0.5. Thereafter, we studied the differentially expressed proteins in both groups. For data mining, we only used the main isoforms of proteins that were directly related to the official gene symbol via UniProt.

### Proteome functional analysis

The 67 differentially expressed proteins were annotated according to UniProt database: UniProt name, gene name, peptide value, coverage, peptide sequence and protein intensity for both groups (via the UniProtKB, human version 2023_03, https://www.uniprot.org/). Statistical analysis was carried out within proteome discoverer using the default multiple test correction (Benjamini–Hochberg). Significance was set via a P-Benjamini–Hochberg (BH) < 0.05.

Functional regulatory network analysis of these proteins was performed using web-based tool STRING (Search Tool for the Retrieval of Interacting Genes/Proteins, version 11.5, http://string.embl.de/).

### Statistical analysis: proteomics

Statistical analyses including independent t-test and chi-square test were performed using SPSS 26.0 software. A difference at P < 0.05 was considered significant. The data are presented as the mean ± SD. (The data were normally distributed according to Kolmogorov–Smirnov test).

## Results

### Demographic and clinical characteristics of the patients

Seven healthy controls (5 females) and five chronic smokers (2 females) were included in the study. All patients had cataract as revealed by slit-lamp examination. The mean age of smokers was 62.4 ± 4.9 and it was 58.6 ± 8.5 years for the control group. There was no statistical difference in age among the groups. Mean ± SD pack-years was 45 ± 27.3 (range 4–80) (Table 1).

**Table 1:** Demographic characteristics of patients, systemic and ocular comorbidities, current medications and the number of packs per year for smokers.

Number of patients	Age/gender	Systemic/ocular co-morbidities	Current medications	Pack/years
Control				
1	49 F	–	–	–
2	52 F	–	–	–
3	61 M	–	–	–
4	52 M	Hypothyroidism Vitrectomy for retinal detachment 28 years earlier	Levothyroxine	–
5	58 F	–	–	–
6	73 F	–	–	–
7	65 F	–	Acetylsalicylic acid discontinued one week before operation	–
Smokers (samples 11, 13 belong to the same patient, however they were obtained at different time points, each from one eye)				
8	65 M	–	–	45
9	60 F	–	–	45
10	56 F	Gastritis Subretinal scar temporal to fovea secondary to trauma in childhood	Omeprazole	4
11	69 M	Hypertension	Amlodipine	54
12	62 M	–	–	80
13	69 M	Hypertension	Amlodipine	54

327 proteins were identified in AH in both groups. Upregulation was defined as abundance ratio with a P value between the smokers’ group and the control group greater than 2 and downregulation was defined as lower than 0.5. According to this definition, 30 proteins were downregulated and 37 proteins were upregulated among smokers (Tables 2, 3).

**Table 2:** Down-regulated proteins among smokers, identified by Student’s t test for unpaired samples (p < 0.05).

#	UniProt AC	Gene symbol	Protein name	Adjusted p-value	Abundance ratio (smokers/controls)	Number of peptides	Molecular mass	Sequence coverage (%)
1	P00751	CFB	Complement factor B	9.00493E−17	0.01	41	85.5	51
2	P98160	HSPG2	Basement membrane-specific heparan sulfate proteoglycan core protein	9.00493E−17	0.01	52	246.3	37
3	P04432	IGKV1D-39	Immunoglobulin kappa variable 1D-39	9.00493E−17	0.01	3	11.5	51
4	A0A2U8J8U9	IgH	Ig heavy chain variable region	9.00493E−17	0.01	3	10.2	28
5	P06310	IGKV2-30	Immunoglobulin kappa variable 2–30	9.00493E−17	0.01	4	12.5	42
6	P01036	CST4	Cystatin-S	9.00493E−17	0.01	4	16.2	34
7	Q02985	CFHR3	Complement factor H-related protein 3	9.00493E−17	0.01	3	37.3	11
8	P09228	CST2	Cystatin-SA	9.00493E−17	0.01	3	16.4	24
9	P02808	STATH	Statherin	9.00493E−17	0.01	3	7.3	55
10	A0A2U8J8T1	IgH	Ig heavy chain variable region	2.12062E−07	0.189	3	11	25
11	A0A024RDJ0	SPP1	Secreted phosphoprotein 1	1.29309E−05	0.242	10	32.3	51
12	P01602	IGKV1-5	Immunoglobulin kappa variable 1–5	1.45564E−05	0.244	5	12.5	47
13	A0A140CTX8	CRYGS	Gamma-crystallin S	5.2553E−05	0.266	14	21	78
14	A0A286YEY4	IGHG2	Immunoglobulin heavy constant gamma 2	0.000180226	0.289	26	43.8	59
15	P04259	KRT6B	Keratin, type II cytoskeletal 6B	0.000197093	0.291	43	60	60
16	Q6UXB8	PI16	Peptidase inhibitor 16	0.000229546	0.295	4	45.7	11
17	Q6N095	IGHG1	Uncharacterized protein	0.000494137	0.312	30	52.3	47
18	P13671	C6	Complement component C6	0.000721834	0.322	22	104.6	30
19	P15121	AKR1B1	Aldo–keto reductase family 1 member B1	0.000943768	0.329	4	35.8	21
20	P68371	TUBB4B	Tubulin beta-4B chain	0.001944834	0.349	3	49.8	8
21	Q9NZP8	C1RL	Complement C1r subcomponent-like protein	0.002268222	0.354	5	48	17
22	P43320	CRYBB2	Beta-crystallin B2	0.002858824	0.361	21	23.4	85
23	Q65ZC9	scFv	Single-chain Fv	0.003620121	0.368	6	25.6	20
24	A0A0X8GLL6	CRYGC	Gamma-crystallin C	0.004843464	0.378	6	20.9	52
25	P06310	IGKV2–30	Immunoglobulin kappa variable 2–30	0.010276384	0.407	4	13.2	41
26	P80748	IGLV3–21	Immunoglobulin lambda variable 3–21	0.012012861	0.413	4	10.4	78
27	E9PFZ2	CP	Ceruloplasmin	0.015886116	0.425	54	108.8	67
28	P02768	ALB	Albumin	0.030984183	0.461	94	59.5	90
29	P16152	CBR1	Carbonyl reductase [NADPH] 1	0.03219306	0.463	5	30.4	30
30	P11216	PYGB	Glycogen phosphorylase, brain form	0.045869048	0.482	4	96.6	6

**Table 3:** Up-regulated proteins among smokers, identified by Student’s t test for unpaired samples (p < 0.05).

#	UniProt AC	Gene symbol	Protein name	Adjusted P-value	Abundance ratio (smokers/control)	Number of peptides	Molecular mass	Sequence coverage (%)
1	P01857	IGHG1	Immunoglobulin heavy constant gamma 1	0.039107417	2.561	30	52.8	51
2	A0A2U8J906	IgH	Ig heavy chain variable region	0.030984183	2.638	4	10.7	33
3	P0C0L4	C4A	Complement C4-A	0.029341173	2.676	22	30.4	72
4	A0A286YFJ8	IGHG4	Immunoglobulin heavy constant gamma 4	0.016733482	2.837	15	43.8	49
5	A0A024R825	CA3	Carbonic anhydrase	0.016349406	2.844	6	29.6	39
6	P01619	IGKV3-20	Immunoglobulin kappa variable 3–20	0.005864265	3.14	4	11.6	47
7	P15169	CPN1	Carboxypeptidase N catalytic chain	0.004420475	3.232	6	52.3	22
8	A0A087WX77	NCAM1	Neural cell adhesion molecule 1	0.000752801	3.758	10	94.5	16
9	P01780	IGHV3-7	Immunoglobulin heavy variable 3–7	0.000617889	3.817	4	13	38
10	H0YLF3	B2M	Beta-2-microglobulin	4.93722E−05	4.585	4	8.5	51
11	P02042	HBD	Hemoglobin subunit delta	1.74094E−05	4.913	13	16	84
12	U3PXP0	HBA2	Alpha globin chain	1.23647E−06	5.787	4	5.7	76
13	P02760	AMBP	Protein AMBP	8.98424E−07	5.896	3	5.7	75
14	P00915	CA1	Carbonic anhydrase 1	1.50926E−09	8.28	12	28.9	53
15	Q5XTR9	HBD	Hemoglobin delta-beta fusion protein	1.61326E−10	9.207	4	3.9	100
16	P02765	AHSG	Alpha-2-HS-glycoprotein	1.56361E−12	11.3	8	27.3	40
17	P01876	IGHA1	Immunoglobulin heavy constant alpha 1	6.65068E−14	12.87	16	53.3	42
18	Q6VFQ6	HBB	Hemoglobin beta chain	6.65068E−14	12.876	5	4.5	100
19	P68871	HBB	Hemoglobin subunit beta	2.56456E−14	13.391	15	16	90
20	Q6U2E7	C4B	C4B1	2.17531E−14	13.451	3	6.3	77
21	A0A1S5UZ39	HBA2	Hemoglobin subunit alpha	9.00493E−17	21.608	12	20.1	88
22	D6RF35	GC	Gc-globulin	9.00493E−17	41.345	34	53	66
23	P01857	IGHG1	Immunoglobulin heavy constant gamma 1	9.00493E−17	100	30	36.1	72
24	A0A385HVZ2	HBA2	Mutant hemoglobin subunit alpha 2	9.00493E−17	100	11	15.3	87
25	P69905	HBA1	Hemoglobin subunit alpha	9.00493E−17	100	8	10.8	76
26	E7ERK6	CLU	Clusterin	9.00493E−17	100	14	23.7	41
27	D3DP16	FGG	Fibrinogen gamma chain, isoform CRA_a	9.00493E−17	100	20	37.7	50
28	A0A1L7B5J3	SERPINA1	Alpha-1-antitrypsin short transcript variant 1C4	9.00493E−17	100	6	5.1	100
29	A0A0U1RQL8	GSN	Macrophage-capping protein	9.00493E−17	100	8	26.3	43
30	O43505	B4GAT1	Beta-1,4-glucuronyltransferase 1	9.00493E−17	100	7	21.9	59
31	P01880	IGHD	Immunoglobulin heavy constant delta	9.00493E−17	100	7	52.9	23
32	Q8TCZ8	APOE	Apolipoprotein E	9.00493E−17	100	3	6.7	54
33	P04003	C4BPA	C4b-binding protein alpha chain	9.00493E−17	100	6	67	13
34	P0DJI9	SAA2	Serum amyloid A-2 protein	9.00493E−17	100	3	13.5	35
35	P01619	IGKV3-20	Immunoglobulin kappa variable 3–20	9.00493E−17	100	3	11.9	30
36	A0A2Y9CYE9	IgH	Ig heavy chain variable region	9.00493E−17	100	3	11.5	19
37	Q8TF66	LRRC15	Leucine-rich repeat-containing protein 15	9.00493E−17	100	3	64.4	4

### Functional analysis of the identified proteins

**Figure 1:**
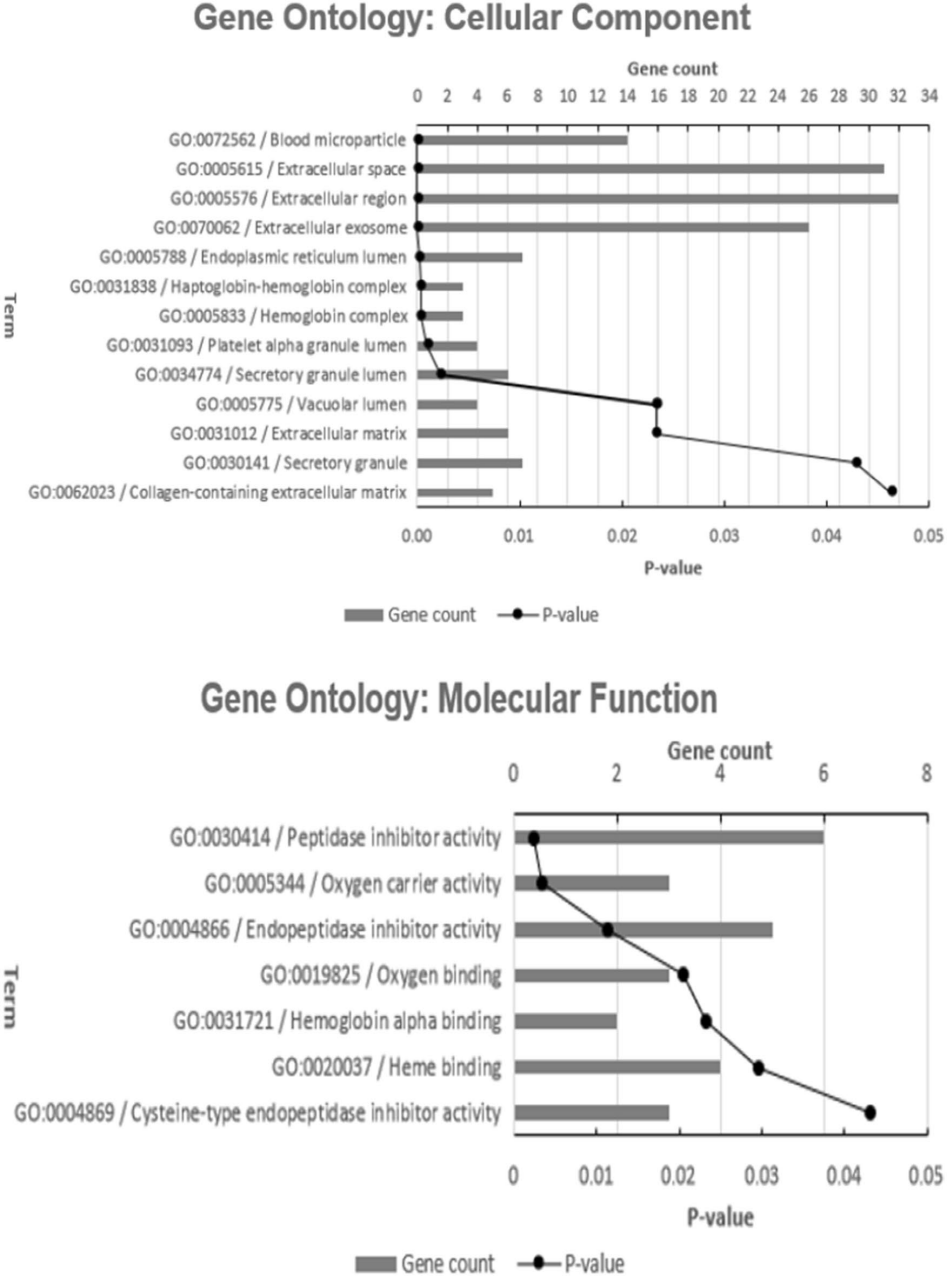

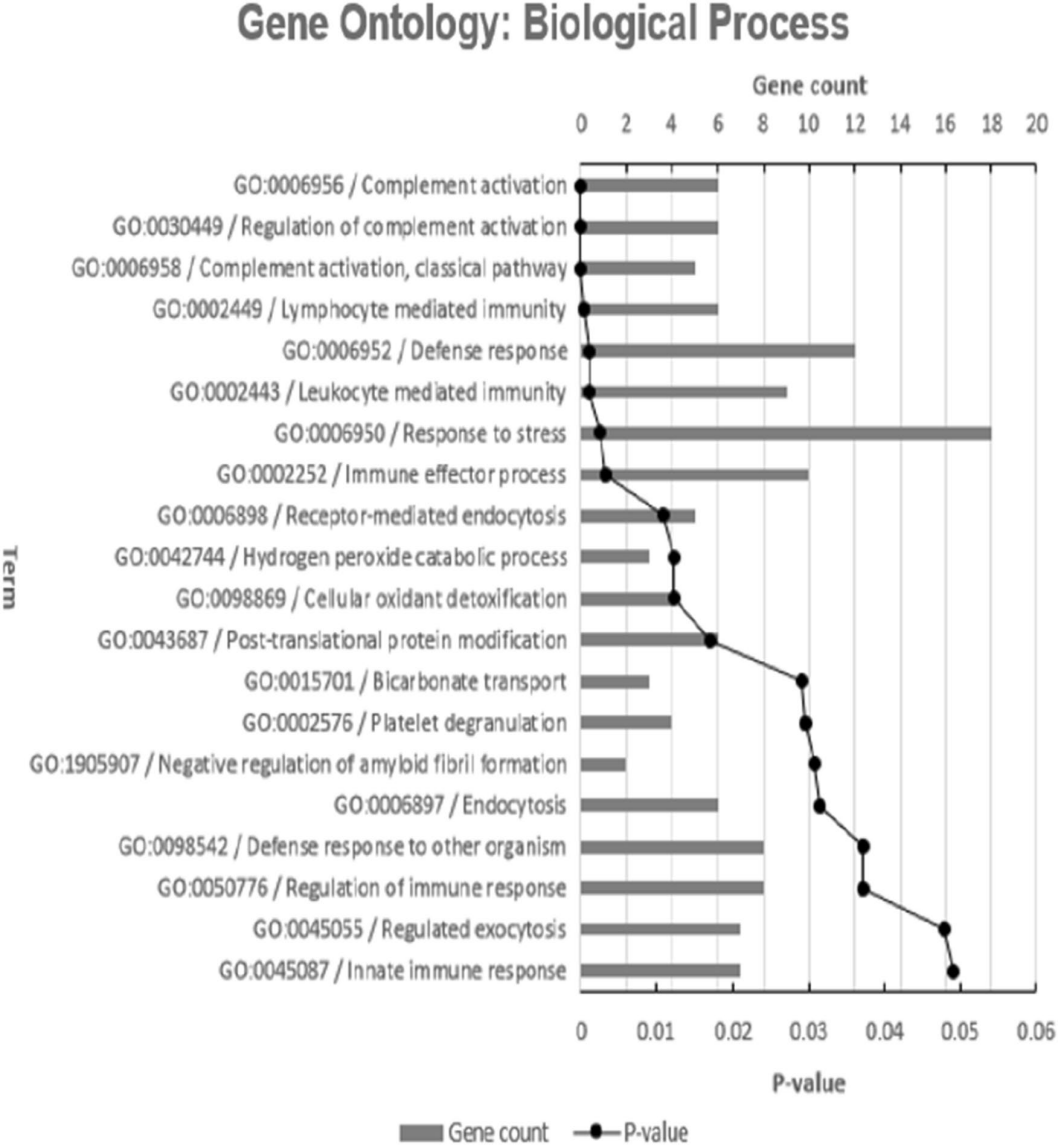
Gene ontology annotation of the proteins identified by STRING. The distribution of identified proteins according to their: cellular component, molecular function and biological process.

GO annotation analysis showed that the 67 proteins can be functionally divided into three groups: cellular component, molecular function, and biological process (Fig. 1). For cellular component ontology, there were 13 GO terms annotated, including extracellular space (GO:0005615; P-value = 1.74E−19) and haptoglobin-hemoglobin complex (GO:0031838; P-value = 0.0003). For molecular function ontology, 7 GO terms were annotated, including peptidase inhibitor activity (GO:0030414; P-value = 0.0024), endopeptidase inhibitor activity (GO:0004866; P-value = 0.0113), cysteine-type endopeptidase inhibitor activity (GO:0004869; P-value = 0.0431), oxygen carrier activity (GO:0005344; P-value = 0.0035) and hemoglobin alpha binding (GO:0031721; P-value = 0.0232). For biological process, a total of 20 GO terms were annotated, including complement activation (GO:0006956; P-value = 8.5E−7), lymphocyte mediated immunity (GO:0006950; P-value = 0.00032), cellular oxidant detoxification (GO:0098869; P-value = 0.0123), bicarbonate transport (GO:0015701; P-value = 0.029) and innate immune response (GO:0045087; P-value = 0.0491).

## Discussion

In this study, we used the comparative proteomics technology to demonstrate the characteristic alterations of the AH proteins in chronic smokers vs healthy controls. The expression levels of 67 protein spots, which were identified by mass spectrometry analysis, showed significant changes in chronic smokers compared with controls. Proteins related to oxidative and cellular defense mechanisms were the most common. Recent advances in proteomic techniques make it possible to monitor protein expression profiles providing a better insight into the mechanisms involved in functional adaptations of cells. This approach allows identifying dysregulated proteins as well as the underlying signaling involved. The advantage of a proteomic rather than a transcriptomic approach is that protein expression levels are measured directly, rather than being inferred from abundance of the corresponding mRNAs, which are imperfectly correlated to protein concentration^46,47^ because of variable rates of synthesis and differences in message stability^48^.

Taken together, our findings are consistent with previous studies and expand current knowledge by providing information of AH biomarkers in chronic smokers, which could be eventually involved in the pathogenesis of ocular pathologies such as AMD in such patients.

Several proteins that are acute phase reactant proteins (APRP) were overexpressed in the AH of chronic smokers. They include Alpha-2-HS-glycoprotein, Clusterin, Fibrinogen, Alpha-1-antitrypsin, Serum amyloid A-2 protein and C4b-binding protein.

Clusterin (CLU) is one of the proteins that was found to be elevated in AH of chronic smokers in comparison to controls. It is also called apolipoprotein J. It is a heterodimeric protein, a member of the small heat shock protein family and, thus, a molecular chaperone, associated with the clearance of cellular debris and apoptosis^49^. A number of studies showed that it was implicated in aging and age-related diseases as neurodegeneration, diabetes, AMD and atherosclerosis^50–52^. It acts as a biomarker of cellular senescence and oxidative stress^53^.

Several studies reported that CLU was upregulated in ocular fluid samples of AMD patients. Kim et al. reported a high level of CLU in AH samples of neovascular AMD (nvAMD) patients compared to controls^54^. Nobl et al.^43^ indicated an increased level of CLU in vitreous samples of patients with nvAMD as compared to controls. On the other hand, Rinsky et al.^44^ demonstrated an increased CLU level in AH samples of patients with atrophic AMD but not in nvAMD.

Smoking is the strongest modifiable risk factor for AMD^55,56^. The macular photocoagulation study^57^ showed that individuals who pursued smoking were at increased risk of recurrent choroidal neovascularization in comparison to nonsmokers in the first year after successful photocoagulation.

CLU was identified in the retinal pigment epithelium (RPE) of AMD donor eyes^58,59^. Yu et al.^60^ aimed to determine effect of cigarette smoke extract on primary human RPE cells. Exposure to 8% of cigarette smoke extract markedly increased mRNA expression of Apo J, CTGF, and fibronectin by approximately three to fourfold and increased the protein expression of Apo J and CTGF and the secretion of fibronectin and laminin.

Yanni et al.^61^ reported that CLU expression density on carotid tissue in patients who underwent endarterectomy was significantly higher in smoking subjects than in non-smoking ones.

Carnevali et al.^62^ found that exposure of cultured human lung fibroblasts to cigarette smoke resulted in a marked accumulation of CLU, both in its precursor form (60 kD) and in its secreted form (76–80 kD). After accumulating within the cells, the 76–80-kD form was released into the incubation medium, where it appeared to protect lung fibroblasts against cigarette smoke-induced oxidative stress.

Fibrinogen was higher in AH of chronic smokers compared to non-smokers. It is a glycoprotein complex that circulates in the blood of vertebrates. During tissue injury, it is converted enzymatically by thrombin to fibrin and then to a fibrin-based blood clot. Fibrin clots function primarily to occlude blood vessels to stop bleeding. It is therefore a prothrombotic component of the hemostatic balance. Fibrinogen is a “positive” acute-phase protein, i.e. its blood levels rise in response to systemic inflammation, tissue injury, and certain other events. Exposure to cigarette smoke is associated with an elevated fibrinogen level and this has been identified as the most potent environmental mediator of an increased plasma concentration of fibrinogen^63–65^. Elevation of fibrinogen by cigarette smoking was reported in studies such as the Ludwigshafen Risk and Cardiovascular Health (LURIC) study and the Coronary Artery Risk Development in Young Adults (CARDIA) study and the Atherosclerosis Risk in Communities (ARIC) study^66–68^. A dose–response relationship has been noted for the number of cigarettes smoked per day and the circulating fibrinogen level^69,70^. Also, the magnitude of increase in fibrinogen levels induced by waterpipe smoking was higher than that induced by cigarette smoking and in the waterpipe group, it was higher in the smokers with more than 3 years of use^71^. The mechanism underlying the association between smoking and an increased fibrinogen level might be related to the chronic proinflammatory state in smokers, which results in a sustained acute-phase response^72^. In smokers, an increased fibrinogen level has been correlated with spontaneous formation of platelet aggregates^73^. The increased level of circulating fibrinogen has been associated with the occurrence of more fibrin-rich thrombi in smokers^74^.

C4b-binding protein (C4BP) was also found to be elevated in AH of chronic smokers in the index study. It is an acute phase protein. Upon inflammation, expression of a form of C4BP composed of exclusively α-chains is increased^75^. It was found to be elevated in smokers. It forms a complex with protein S (which serves as a cofactor of coagulation inhibitor, protein C). The resultant decrease in free protein S and the sequential down-regulation of the protein C pathway could enhance the thrombotic sequelae of smoking^76^. C4BP is an important soluble inhibitor of the lectin and the classical pathways of complement^77^. Its inhibitory effect is exerted by binding to the activated complement component C4b and limiting its the function, thus inhibiting the formation of the C4bC2a complex i.e., classical C3-convertase^78–80^. It also hastens the C3 convertase natural decay^81,82^.

Serum amyloid A-2 protein is another acute phase reactant protein found to be elevated in AH of chronic smokers. There are 4 SAA genes in humans of which SAA1 and SAA2 encode acute phase proteins. A multitude of tissues express SAA such as breast, brain neurons, intestine and stomach^83,84^. SAA has many pro-inflammatory and pro-atherogenic activities. The levels of SAA are markedly linked with blood coagulability^85,86^ though induction of agglutination of red blood cells, activation of platelets and their subsequent clumping^87^.

Al-Sieni et al. showed significant increase in SAA in correlation to the degree of smoking in particular in the age category older than 40 years of age^88^. Circulating SAA is preferentially associated with HDL. Wilson et al.^89^ recently reported that SAA can be exchanged between HDL and VLDL/LDL. SAA–LDL complex reflects oxidatively modified LDL particles^90^. A significantly higher SAA-LDL levels were observed in current smokers versus non-smokers^91^.

Alpha 1-antitrypsin (ATT) is another acute phase protein^92–95^ found to be elevated in AH of chronic smokers. In smokers, the levels of ATT were exclusively and considerably raised and were related to the extent of smoking^96^. It plays an important role in protecting the lungs from the neutrophil elastase enzyme that disrupts the connective tissues. ATT is the most abundant endogenous serine protease inhibitor in the blood^95^. Besides its antiprotease activity, AAT possesses anti-inflammatory activity against lymphocytes, neutrophils, macrophages^97^ and against several of the pro-inflammatory cytokines such as TNF-α, IFN-γ and IL-1-β^98–101^. It has antiapoptotic effect in other cells^102–109^.

Alpha-2-HS glycoprotein, an acute phase reactant protein, was found to be elevated in AH of chronic smokers. It is secreted predominantly by the liver^110^, however other extrahepatic tissues can produce it^111,112^. It may work as a positive or negative acute phase protein depending on the mode of stimulation. It acts as a protective agent in severe systemic inflammation^113,114^. Chang et al.^115^ reported on the AH protein expressions in patients who underwent cataract operation. Patients had cataract risk factors such as DM and smoking whereas the control group had cataract but no other risk factors. The expression of alpha-2-HS-glycoprotein was increased in the presence of risk factors. The authors thus suggested that it could be a potential aqueous biomarker associated with DM and smoking.

APRPs show a rapid response to inciting events such as inflammation, infection, surgery and myocardial infarction. The acute-phase response may be short-lasting as with a transient infection, or it can be long-lasting as in the set-up of chronic conditions^116^. These processes usually exert inflammation aiming to eliminate tissue debris and enhance tissue repair^117^. The magnitude of this response is related quantitatively to the activity or extent of inflammation in the acute situation. APRPs are normally synthesized by the liver and this is regulated by cytokines especially interleukin-6 (IL-6). Cytokines are produced by cells involved in the inflammatory cascade such as macrophages and endothelial cells^118^. These cytokines may induce small to very significant changes in APRP levels, which usually increase simultaneously, however not uniformly even in patients with the same inflammatory condition.

Smoking enhances systemic inflammation by augmenting the release of inflammatory cells into the circulation and by increasing the levels of pro-inflammatory cytokines (such as TNF, IL1, IL6, IL8) and APRPs^119^. Cigarette smoke was also found to decrease the levels of anti-inflammatory cytokines such as IL-10^120^. Wannamethee et al.^121^ examined the association between cigarette smoking and inflammatory markers in 2920 British men. It was demonstrated that current smokers had higher levels of the CRP and white blood cell (WBC) count compared with never smokers. Petrescu et al.^122^ measured serum levels of the key pro-inflammatory player TNF-α in healthy heavy smokers and nonsmokers. Serum levels of TNF-α were markedly higher in smokers. The study showed that there was a positive correlation between TNF serum levels and the extent of exposure to tobacco smoke. IL-1 ß serum level was also higher in active smokers in comparison to nonsmokers^123^. Both cytokines IL-1 ß and TNF-α play major roles in the pathogenesis of inflammatory disorders^123^.

As observed in Fig. 1, the molecular function of the involved proteins was related to oxygen carrier activity, oxygen binding and hemoglobin alpha binding. Among the upregulated proteins was hemoglobin subunit delta (HBD), hemoglobin subunit beta (HBB) and hemoglobin subunit alpha (HBA2 and HBA1). HbA is the most common form of hemoglobin in adults, consisting of two α chains and two β chains (α2 β2). Hemoglobin (Hb) is the iron-containing oxygen-transport metalloprotein in the red blood cells. In the blood, it carries oxygen from the lungs to the rest of the body. Hb levels are significantly higher for smokers than for never-smokers and this is related to the number of cigarettes smoked daily^124^. The increase in Hb level among smokers is largely related to increased levels of carboxyhemoglobin (HbCO) which is an inactive form of Hb resulting from exposure to carbon monoxide^125^.

Cellular oxidant detoxification was among the biological processes noted in Fig. 1. Alpha-1-microglobulin/bikunin precursor (AMBP) is a relevant protein in this context, that was upregulated in the index study. After proteolytic cleavage, A1M and bikunin are secreted into the blood as separate proteins^126^.

A1M is a ubiquitous protein with reductase and radical- and heme-binding properties. It continuously removes free radicals and oxidizing agents, particularly heme, from the tissues. It is subsequently transported to the kidneys, where it is broken down. The protein is therefore believed to protect cells and tissues against the damage that is induced by abnormally high concentrations of free hemoglobin and/or reactive oxygen species^127^.

Of the downregulated proteins were those that belong to the crystallin lens family including Gamma-crystallin S (CRYGS), Beta-crystallin B2 (CRYBB2) and Gamma-crystallin C (CRYGC). Crystallins are the major proteins in the lens and lens clarity derives from crystallins—abundant water-soluble proteins. Crystallins produce a gradient of refractive index from the center to the periphery of the lens^128^. Three classes of “classical” crystallins are present in vertebrate lenses: α, ß, and Ɣ. In-vivo functions of ß B2-crystallin were examined via the generation of mice with a targeted disruption of Crybb2. The lens appeared to develop normally in the first months of life. In older animals, the weight and axial diameter of the lenses of knockout mice were significantly smaller than in wild-type mice. Cataracts were formed in the posterior and anterior cortex several months after birth and cataract severity increased with age. The knockout lenses also showed decreased resistance to oxidative stress^129^.

This study has several limitations. First, the sample size was small. Second, there were more female subjects among healthy controls than among chronic smokers. As a result, the bias of the gender influencing the proteomic profile cannot be excluded. Therefore, the comparisons are preliminary. There was no difference in the age groups of chronic smokers and nonsmokers. This may imply that age-related changes in the proteomic profile can be ruled-out in the index study. Another limitation was that two samples of the smokers group belonged to the same patient, however the second sample was obtained 7 months later, it therefore did not represent an identical biological replicate to the patient’s first sample.

On the other hand, the study is among the first studies to report on the proteomic profile of AH among chronic smokers. The subjects in the control group and chronic smokers were carefully selected. Patients with common systemic conditions such as diabetes mellitus and hyperlipidemia were excluded, as were the patients on chronic anti-inflammatory medications. The differential expression of APRPs in the AH of chronic smokers in our study is an addition to the existing knowledge on the systemic alterations triggered by smoking. This may further promote the understanding of the molecular pathways involved and would add more information on the significance of APRPs in ocular proteomics.

## Data Availability

The mass spectrometry proteomics data have been deposited to the ProteomeXchange Consortium via the PRIDE partner repository with the dataset identifier PXD045087.
